# Supplementary material for: T cell perturbations persist for at least 6 months following hospitalization for COVID-19
Source: Front Immunol. 2022 Aug 8;13:931039. doi: 10.3389/fimmu.2022.931039 (PMC9393525; doi:10.3389/fimmu.2022.931039)
Supplement: Supplementary file 1 [file DataSheet_1.docx]

**Supplementary figures and tables Govender et al**

**Supplementary Table 1.**

**Monoclonal antibodies used for quantification of lymphocytes in**

**whole blood**

| Marker | Fluorochrome | Company | Clone |
| --- | --- | --- | --- |
| CD57 | FITC | BD | HNK-1 |
| IgD | FITC | BD | IA6-2 |
| CD27 | PE | BD | L128 |
| CD45 | PerCP | BD | 2DI |
| CD38 | PerCP-Cy5.5 | BD | HIT2 |
| CD19 | PE-Cy7 | BD | SJ25C1 |
| CD56 | PE-Cy7 | BD | NCAM16.2 |
| CD8 | APC | BD | SK1 |
| CD21 | APC | BD | B-ly4 |
| CD20 | APC-H7 | BD | L27 |
| CD4 | Horizon V450 | BD | RPA-T4 |
| CD3 | Horizon V500 | BD | UCHT1 |

Antibodies used at 4µl to 50ul whole blood

**Supplementary Table 2.**

**Antibodies used for T cell characterization**

| Marker | Fluorochrome | Company |  | Clone | Concentration |
| --- | --- | --- | --- | --- | --- |
| CD3 | BV750 | BD |  | SK7 | 1/160 |
| CD45 | APC | BD |  | HI30 | 1/400 |
| CD4 | Spark NIR 685 | BioLegend |  | SK3 | 1/80 |
| CD8 | AF700 | BioLegend |  | SK1 | 1/400 |
| HLA-DR | BV570 | BioLegend |  | L243 | 1/40 |
| CD38 | APC Fire 810 | BioLegend |  | HIT2 | 1/40 |
| CD57 | Pacific blue | BioLegend |  | HCD57 | 1/400 |
| PD1 | PE-Cy5.5 | Novus Biologicals |  | J116 | 1/40 |
| CD45RA | FITC | BD |  | HI100 | 1/200 |
| CCR7 | PE | BioLegend |  | G043H7 | 1/20 |
| CD27 | PE-Cy7 | BioLegend |  | O323 | 1/40 |
| CD28 | PerCP | ThermoFisher |  | CD28.2 | 1/20 |
| CD25 | BV650 | BD |  | M-A251 | 1/40 |
| CD127 | BV785 | BioLegend |  | A019D5 | 1/40 |
| CD95 | BV605 | BioLegend |  | DX2 | 1/40 |
| CXCR5 | BV510 | BD |  | RF8B2 | 1/20 |
| TIM-3 | BV421 | BioLegend |  | F38-2E2 | 1/80 |
| LAG-3 | PE-CF594 | BD |  | T47-530 | 1/40 |
| ICOS | BV711 | BD |  | DX29 | 1/40 |
| CCR6 | PerCP-eFlour 710 | ThermoFisher |  | R6H1 | 1/80 |
| CXCR3 | BV480 | BD |  | 1C6 | 1/20 |
| CD69 | APC-H7 | BD |  | FN50 | 1/40 |
| Viability | ViaKrome 808 | Beckman Coulter |  |  | 1/50 |

**
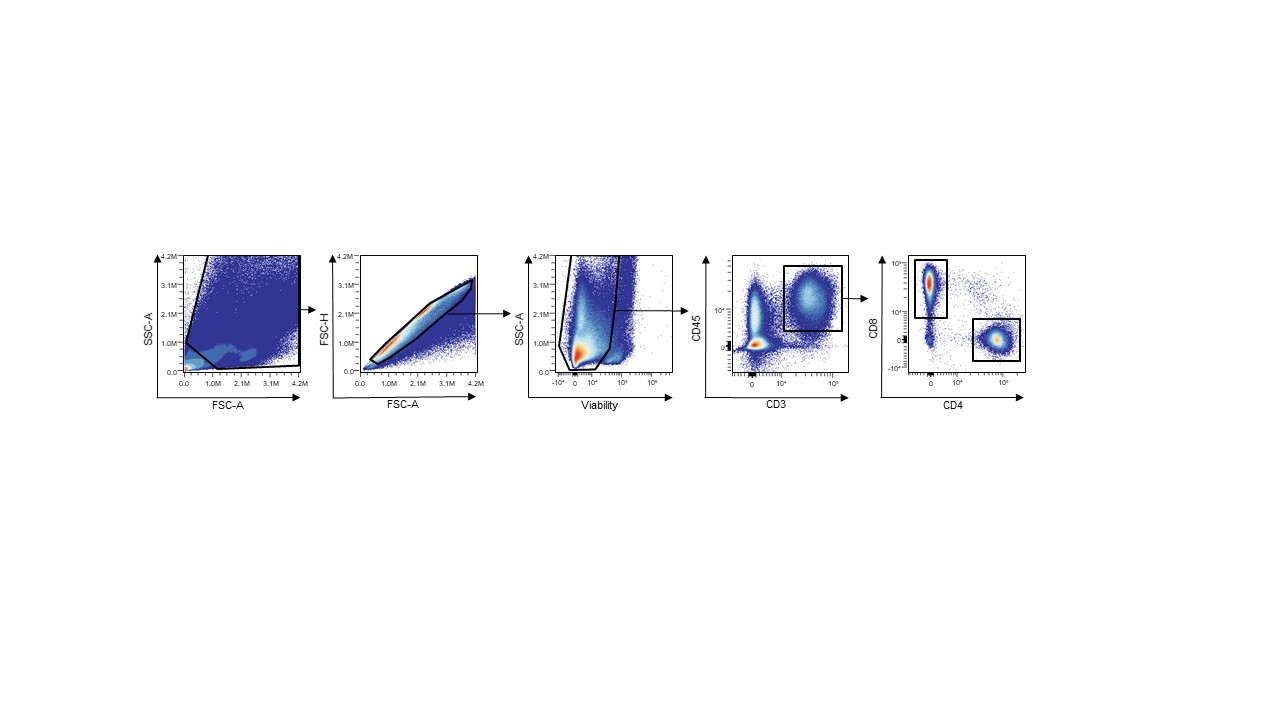
Supplementary Figure 1.**

**Gating strategy to define bulk and CD4^+^ and CD8^+^ T cells.**

Suspensions of PBMCs were prepared by blocking and antibody staining for analysis by spectral flow cytometry. Representative gating strategy is shown here, depicting how CD4^+^ and CD8^+^ lymphocytes were selected for further analysis, using ViaKrome808 viability dye, CD3, CD45, CD4 and CD8 monoclonal antibodies.


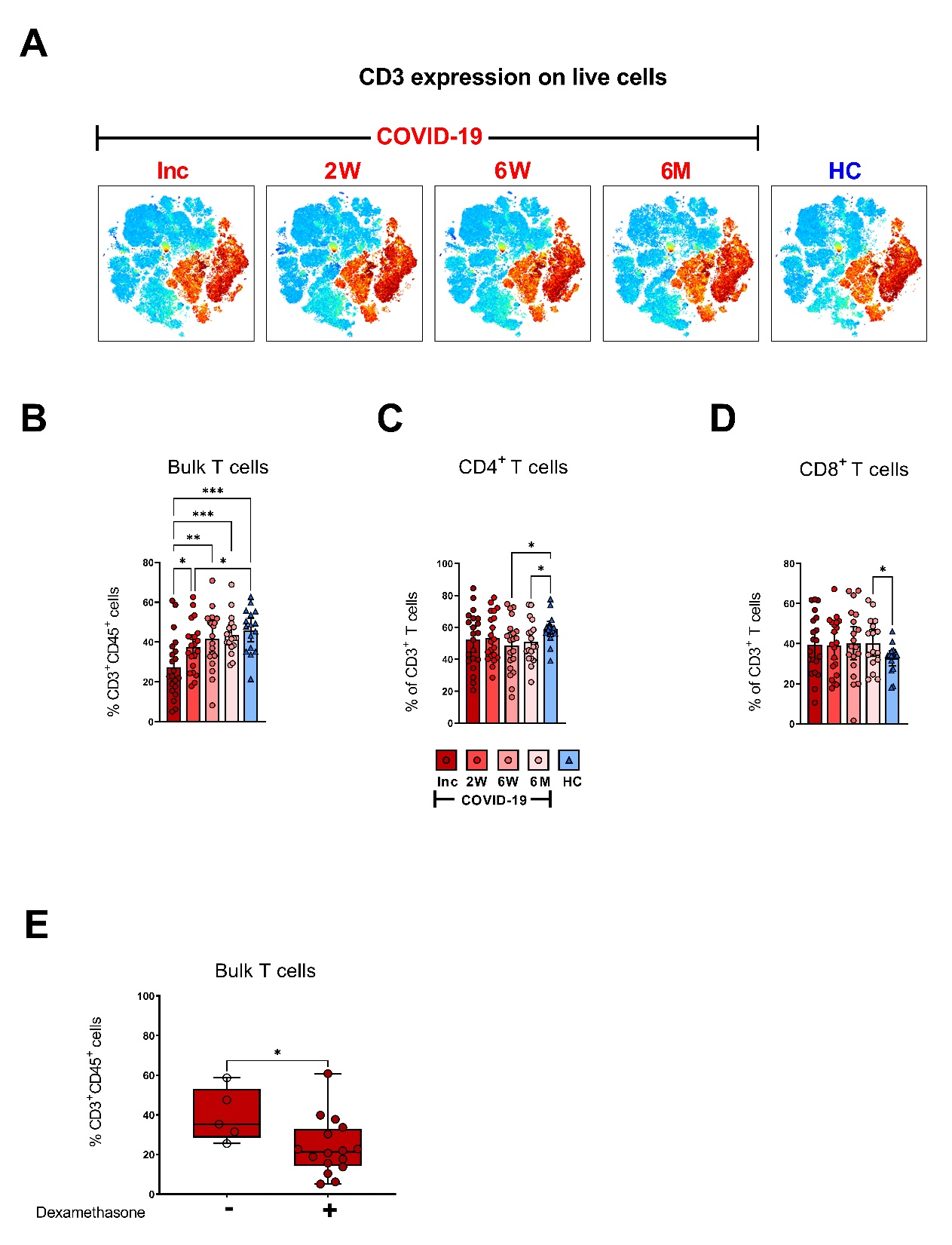


**Supplementary Figure 2.**

**Bulk, CD4^+^ and CD8^+^ T cells during COVID-19 infection.**

PBMCs obtained from 23 COVID-19 patients and 16 healthy donors over a 6-7 month period were stained with CD45, CD3, CD4 and CD8 monoclonal antibodies and assessed by flow cytometry. (**A**) tSNE plots show live cells, after removal of debris and dead cells, colored according to intensity of CD3 expression. Measurements of the major T cells subsets: (**B**) CD3^+^, (**C**) CD4^+^ T cells and (**D**) CD8^+^ T cells as shown in percentages. (**E**) Percentage of CD3^+^ T cells in COVID-19 patients with or without dexamethasone. CD3^+^ T cells Data is represented as mean with 95% Cl, with significance of *p ≤ 0.05, **p ≤ 0.01, ***p ≤ 0.001, ****p ≤ 0.0001, determined using Brown-Forsythe and Welch ANOVA tests. Inc = Inclusion in study at the hospital, 2W = 2 weeks, 6W = 6 weeks, 6M = 6-7 months, HC = healthy control.


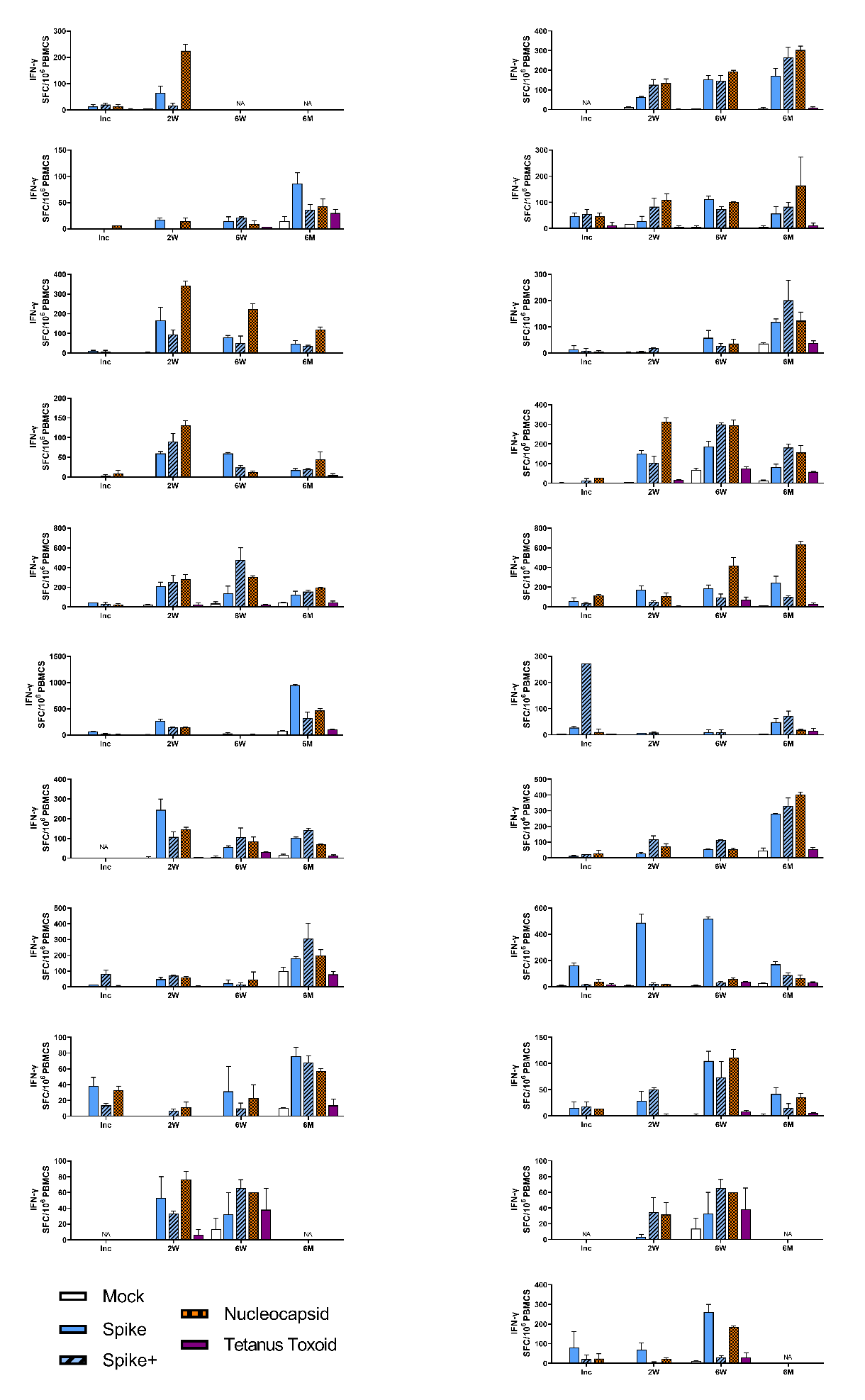
**Supplementary Figure 3.**

**SARS-CoV-2-specific T cells in individual COVID-19 patients.**

PBMCs collected from 21 covid patients were unstimulated (Mock) or stimulated with SARS-CoV-2 specific overlapping peptides from: Spike, spike+, and nucleocapsid and tetanus toxoid protein. The IFN-γ production was measured after 48h as spot forming cells (SFC) by ELISPOT. Each graph represents a single donor. Data is represented as mean with 95% Cl, with significance of **p<0.01, ***p < 0.001, ***p < 0.0001 determined using Brown-Forsythe and Welch ANOVA tests.
